# Lamin B1 in cancer and aging

**DOI:** 10.18632/aging.102306

**Published:** 2019-09-20

**Authors:** Boyan K. Garvalov, Sajjad Muhammad, Gergana Dobreva

**Affiliations:** 1 Department of Microvascular Biology and Pathobiology, European Center for Angioscience (ECAS), Medical Faculty Mannheim, Heidelberg University, Mannheim 68167, Germany; 2 Department of Anatomy and Developmental Biology, Centre for Biomedicine and Medical Technology Mannheim (CBTM) and European Center for Angioscience (ECAS), Medical Faculty Mannheim, Heidelberg University, Mannheim 68167, Germany

**Keywords:** lamin B1, lung cancer, RET, epigenetic regulation, aging, senescence

The nuclear lamina is a network of intermediate filament proteins called lamins that play a central role in regulating the shape and organization of the nucleus. Two types of lamins exist, A-type and B-type, whose functions encompass the control of nuclear structure, chromatin organization, gene positioning, DNA replication and repair, cell cycle progression, stress responses, proliferation and differentiation. A- and B-type lamins form distinct, but interacting filament meshworks, so that changes in one type of lamin can also alter the organization and activity of the other [1]. Furthermore, lamins interact with a variety of binding partners that mediate their diverse cellular functions, including association to specific regions of chromatin: while A-type lamins bind both hetero- and euchromatin, B-type lamins interact with relatively gene-poor and transcriptionally inactive domains. Changes in lamins have a strong link to aging: for example, mutations in lamin A lead to the premature aging diseases Hutchinson-Gilford progeria syndrome and atypical Werner syndrome [1]. Lamins are also implicated in a broad range of other pathologies, including aging-associated diseases such as cardiovascular morbidities and cancer.

In our recent work, we focused on the pathophysiological role of lamin B1 [2]. While complete loss of Lamin B1 is embryonically lethal, we found that mice with hemizygous deletion of *Lmnb1* showed a strikingly increased incidence of lung tumor formation. Based on these observations, we proceeded to characterize the function of lamin B1 in the development of lung cancer, the leading cause of cancer-related death. We observed that lamin B1 levels were reduced in lung cancer patients compared to normal lung tissue and that lower expression of lamin B1 was associated with higher tumor grade. The levels of lamin A, on the other hand, were not altered in human lung tumors, indicating that the different types of lamins have distinct functions in lung carcinogenesis. We further demonstrated that depletion of lamin B1 in lung epithelial cells greatly increased their anchorage-independent growth and migration *in vitro*, as well as their tumorigenic and metastatic capacity *in vivo*.

**Figure 1:**
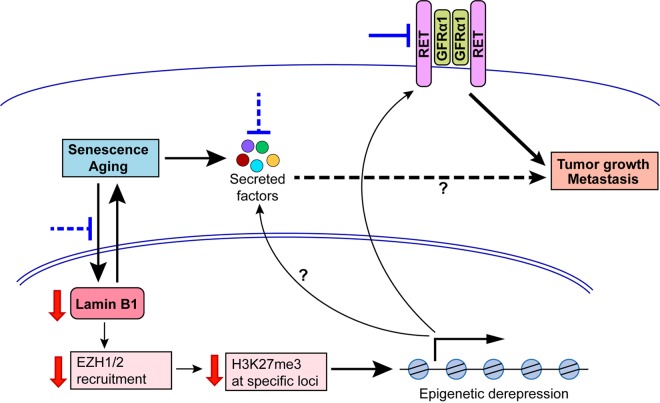
**A model of the function of lamin B1 in the control of lung tumorigenesis and its potential link to aging/senescence.** Lamin B1 recruits EZH1/EZH2 to catalyze H3K27me3 and repress the expression of genes, including RET and GFRα1. When lamin B1 is depleted, EZH1/2 recruitment and H3K27me3 marks are reduced, RET/GFRα1 are upregulated and promote lung tumor growth and metastasis. Interference with Ret signaling using available inhibitors (blue blunted arrows) could suppress the growth and progression of lamin B1-deficient lung tumors. Furthermore, aging and cellular senescence are linked to a decrease of lamin B1 in older tissues. Loss of lamin B1 can induce a senescent state that may in turn be linked to certain aspects of tumor progression through secreted factors produced by senescent cells. Such factors might be regulated by lamin B1-EZH1/2-dependent changes in gene expression. Counteracting mechanisms linking senescence to lamin B1 and vice versa may therefore also hamper cancer development and metastasis (blue dashed blunted arrows).

Lamin B1 has been shown to bind specific regions of the genome positioned at the nuclear lamina, which are characterized by a repressive chromatin state and reduced transcription [3]. Consistent with a repressive function of lamin B1 on gene expression, we observed that most of the differentially expressed genes were upregulated after lamin B1 loss. Among the most prominently upregulated genes was the receptor tyrosine kinase RET, a well-established oncogene, and its co-receptor GFRα1. Importantly, the activation of RET played a crucial role in mediating the effects of lamin B1 loss, as silencing of RET in lamin B1-depleted cells blocked migration, tumor growth and metastasis. The clinical relevance of these findings was supported by analysis of human lung tumor samples that showed an inverse correlation between the levels of lamin B1 and RET. Although lamin B1 has been associated with repressive chromatin and decreased transcription, it has so far remained unclear if lamin B1 plays a direct functional role in mediating chromatin modification and consequent changes in gene expression. Indeed, while in some cases gene positioning at the nuclear periphery leads to changes in gene activity in other cases it does not. Our studies demonstrated that not only lack of tethering to the nuclear periphery but also loss of chromatin binding of the histone methyltransferases EZH1 and EZH2, which catalyze methylation of the repressive histone mark H3K27me3, was essential for activation of the Ret gene. EZH1 appears to play a more prominent role, since its silencing phenocopied the effect of lamin B1 depletion on the malignant phenotype of lung epithelial cells. Consistently, lung cancer patients expressing low levels of EZH1 had a significantly worse prognosis than patients with higher EZH1 expression. Taken together, our results demonstrate that loss of lamin B1 is a common feature in lung cancers, leading to epigenetic derepression of RET and activation of RET signaling, which can be pharmacologically targeted in lamin B1-deficient tumors (Figure 1).

While our study focused on RET activation upon lamin B1 loss, future work can address the potential involvement of additional lamin B1-dependent mechanisms in tumorigenesis and any links that they may have to aging and senescence. Interestingly, downregulation of lamin B1 is a common feature of senescent cells [4]. This is in line with the fact that tissue-specific lamin B expression decreases with age in *Drosophila* [5] and our unpublished observations that the same happens in mice. Cellular senescence and aging have been linked to distinct chromatin changes, including a large-scale decrease of H3K27me3 marks at specific domains [6]. Importantly, depletion of lamin B1, EZH1 or EZH2 led to a similar induction of senescence with establishment of senescence-like chromatin changes [6,7]. These findings suggest that lamin B1 downregulation and downstream epigenetic alterations mediated by it may be not simply a consequence, but rather a cause of senescence. This could provide an additional link to carcinogenesis: while senescence is classically perceived as a barrier to malignant transformation, it can promote certain aspects of cancer progression, e.g. via factors secreted by senescent cells [8]. Indeed, the genes for most typical components of the senescent secretome are found within H3K27me3-depleted chromatin regions in senescent cells [4,6], suggesting that lamin B1-EZH1-dependent mechanisms might be involved in controlling their expression. This could represent a novel mechanism contributing to the well-known but still insufficiently understood association between aging and increased propensity for tumor development.
